# Analytical method development and validation of a serum LC–MS/MS assay for 2 emerging synthetic cannabinoids: 5F-MDMB-PICA and MDMB-4en-PINACA

**DOI:** 10.1097/MD.0000000000050058

**Published:** 2026-08-14

**Authors:** Murat Akbaba, Aysun Baransel Isir, Süleyman İbili, Can Erdem Yiğitsoy

**Affiliations:** a Department of Forensic Medicine, Faculty of Medicine, Gaziantep University, Gaziantep, Turkey; b Department of Plant Protection, Faculty of Agriculture, Gaziosmanpasa University, Tokat, Turkey.

**Keywords:** 5F-MDMB-PICA, LC–MS/MS, MDMB-4en-PINACA, method validation, serum analysis, synthetic cannabinoids

## Abstract

**Background:**

This study aimed to develop and validate a highly sensitive and reproducible liquid chromatography–tandem mass spectrometry (LC–MS/MS) method for the simultaneous determination of 5F-MDMB-PICA and MDMB-4en-PINACA in human serum, ensuring suitability for both forensic and clinical toxicology applications.

**Methods:**

Drug-free serum samples were fortified with target analytes, extracted through solid-phase extraction, and analyzed using a triple-quadrupole LC–MS/MS system (Shimadzu LCMS-8045) equipped with a biphenyl column. Calibration was established within 1–100 ng/mL, and validation parameters – linearity, sensitivity, accuracy, and precision – were assessed according to international guidelines.

**Results:**

The calibration curves displayed excellent linearity (*r*^2^ = 0.999) for both analytes. The limits of detection were 0.113 ng/mL for 5F-MDMB-PICA and 0.145 ng/mL for MDMB-4en-PINACA, with limits of quantification of 0.339 ng/mL and 0.435 ng/mL, respectively. Recovery rates at 10 and 50 ng/mL ranged from 77.7% to 96.4%, while intra and inter-day precision (relative standard deviation %) remained below 15%.

**Conclusion:**

The validated method provides reliable quantification of 5F-MDMB-PICA and MDMB-4en-PINACA in human serum. The observed linearity, sensitivity, recovery, and precision support its applicability for routine forensic toxicology analyses.

**Key points**

1. A fully validated liquid chromatography–tandem mass spectrometry assay was developed for the quantitative determination of the synthetic cannabinoids *5F-MDMB-PICA* and *MDMB-4en-PINACA* in human serum.
2. The method achieved excellent linearity (*r*^2^ = 0.999) across 1–100 ng/mL, demonstrating strong quantitative performance.
3. High analytical sensitivity was established with low detection limits (LOD: 0.113–0.145 ng/mL) and quantification limits (LOQ: 0.339–0.435).
4. Recovery rates (77.7%–96.4%) and precision values (repeatability and within-laboratory reproducibility <15%) fully satisfied international bioanalytical validation criteria (European Medicines Agency/Food and Drug Administration).
5. The optimized biphenyl chromatographic phase improved isomeric separation and reduced matrix effects compared with conventional C18 columns.
6. The method provides a validated analytical workflow suitable for forensic toxicology laboratories.
7. This study provides a validated serum-based liquid chromatography–tandem mass spectrometry quantification protocol for 5F-MDMB-PICA and MDMB-4en-PINACA and contributes additional analytical data to the emerging literature on synthetic cannabinoids.

## 1. Introduction

The rapid worldwide emergence of new psychoactive substances has created major public health and forensic concerns. Among them, synthetic cannabinoids represent 1 of the most critical subclasses due to their exceptional potency and unpredictable physiological responses. In recent years, 5F-MDMB-PICA and MDMB-4en-PINACA have become particularly noteworthy, as they are frequently identified during toxicological analyses and have been implicated in serious clinical outcomes, including convulsions, cardiac complications, and even fatal intoxications.^[1–6]^

In contrast to phytocannabinoids of natural origin, synthetic analogues exhibit considerable structural diversity, which makes both their detection and quantification technically demanding. Their ability to bind cannabinoid receptor type 1 and cannabinoid receptor type 2 receptors with greater affinity than Δ9-tetrahydrocannabinol results in markedly enhanced psychoactive and toxic effects. Consequently, developing analytical techniques capable of detecting these compounds rapidly and precisely in biological matrices is essential for clinical, forensic, and legal evaluations.^[1,7–9]^

Liquid chromatography–tandem mass spectrometry (LC–MS/MS) has long been regarded as the benchmark analytical tool for identifying synthetic cannabinoids because of its superior sensitivity and selectivity. Its adaptability for complex biological samples such as serum and plasma enables reliable quantification even at trace concentrations. However, the continuous introduction of new cannabinoid analogues necessitates the ongoing optimization and validation of compound-specific analytical procedures. Such validation, in accordance with international recommendations, ensures the method’s accuracy, precision, and reproducibility under variable laboratory conditions.^[10–13]^ In LC–MS/MS protocols, solid-phase extraction (SPE) is commonly integrated to improve analyte recovery, minimize matrix interference, and enhance consistency. Essential performance indicators – linearity, limits of detection (LOD) and limits of quantification (LOQ), recovery, and precision – must therefore be systematically examined to demonstrate the robustness of the analytical workflow.^[14–17]^

Currently, there is no published, fully validated LC–MS/MS protocol for quantifying 5F-MDMB-PICA and MDMB-4en-PINACA in human serum. Given the increasing incidence of synthetic cannabinoid use, there is a pressing need for reliable toxicological methodologies and for expanding the scientific evidence base regarding these compounds. Establishing a sensitive, reproducible, and validated analytical approach is crucial, as it facilitates accurate identification, supports clinical diagnostics, assists in the interpretation of forensic evidence, and contributes to monitoring substance use trends.

Accordingly, the present study was designed as a comprehensive validation project to establish an LC–MS/MS workflow for 5F-MDMB-PICA and MDMB-4en-PINACA in serum. This validated method provides a methodological framework that can be directly applied to forensic and clinical case samples in future investigations.

## 2. Materials and methods

### 2.1. Study design, matrix source, and ethical approval

This study was conducted as a single-center analytical method development and validation study at the Forensic Toxicology Laboratory, Department of Forensic Medicine, Gaziantep University. Drug-free human serum samples were used exclusively as blank biological matrix material and were fortified with certified reference standards of 5F-MDMB-PICA and MDMB-4en-PINACA for method validation purposes.

Ethical approval was obtained from the Clinical Research Ethics Committee of Gaziantep University Faculty of Medicine (approval no: 2023/435; Date: December 30, 2023). Written informed consent was obtained from all volunteer serum donors prior to blood collection. The study was conducted in accordance with the ethical principles of the Declaration of Helsinki and its subsequent amendments.

### 2.2. Sample collection and instrumentation

Whole-blood samples were collected from volunteer laboratory personnel who were confirmed to be free from narcotic or stimulant drug use. Serum obtained from these blank samples was used solely as a negative matrix, fortified post-collection with certified standards of 5F-MDMB-PICA and MDMB-4en-PINACA. No human exposure or pharmacological intervention was performed. Following confirmation of drug-free status, serum was separated and analyzed using a Shimadzu Triple Quadrupole LCMS-8045 system (Shimadzu Corp.). SPE was performed using Oasis HLB 3cc cartridges (60 mg, Waters Corp.). Extraction conditions were optimized using sequential elution with 70% acetone followed by 2% ammonia in ethyl acetate, which yielded higher recovery and cleaner chromatograms compared with alternative sorbents (C18, MCX). Chromatographic analysis was performed using a Restek Raptor Biphenyl column (2.1 × 100 mm, 2.7 μm particle size). The biphenyl stationary phase was selected to enhance selectivity for positional isomers compared with conventional C18 columns.

### 2.3. Reagents and solutions

Standard solutions containing 5F-MDMB-PICA and MDMB-4en-PINACA were prepared in methanol at 7 different concentrations (1, 2.5, 5, 10, 25, 50, and 100 ng/mL). Diazepam-d_5_ was added as a procedural control to monitor extraction performance and instrument stability throughout the analytical process. It was not used for quantitative calibration, response normalization, or calculation of validation parameters. Stable isotope-labeled analogues of 5F-MDMB-PICA and MDMB-4en-PINACA were not available during the study period; therefore, diazepam-d_5_ was employed solely as a procedural control rather than as a quantitative internal standard. Extraction solvents included 5% methanol (v/v), 70% acetone (v/v), and 2% ammonia in ethyl acetate. The mobile phases were prepared as follows: 2 mM ammonium acetate in 0.1% formic acid, methanol containing 0.1% formic acid, and 66.6% methanol. All solutions were degassed in an ultrasonic bath for 10 minutes prior to use.^[18]^

### 2.4. LC–MS/MS conditions

Analyses were carried out under optimized LC–MS/MS conditions. Chromatographic separation on the biphenyl column enhanced isomeric discrimination between 5F-MDMB-PICA and MDMB-4en-PINACA through π–π interactions, reducing matrix effects and improving peak resolution. The mobile phase consisted of methanol and ammonium acetate/formic acid buffer, optimized to minimize serum-derived matrix effects and improve signal-to-noise ratios.

The mobile-phase flow rate was maintained at 0.6 mL/min with a maximum system pressure of 660 bar. The injection volume was 10 μL, and the column temperature was set at 40°C. Ionization was achieved via electrospray ionization using nitrogen as the nebulizing and drying gas. The parameters were adjusted as follows: drying gas temperature, 300°C; drying gas flow, 10 L/min; nebulizing gas flow, 3 L/min; dissolution temperature, 526°C; and heater gas flow, 10 L/min.^[18]^

### 2.5. Preparation of blood samples for calibration

Drug-free serum samples were fortified with known concentrations of analytes to construct calibration curves and perform validation tests. For calibration, 1 mL of serum was mixed with 20 μL of diazepam-d_5_ procedural control solution and 2.5 mL of distilled water, followed by centrifugation at 3500 rpm for 10 minutes. The supernatant was loaded onto SPE cartridges preconditioned with 2 mL of methanol and 2 mL of distilled water. Cartridges were washed with 2 mL of 5% methanol and dried under vacuum for 10 minutes. Elution was performed sequentially with 2 mL of 70% acetone and 2 mL of 2% ammonia in ethyl acetate. The eluates were evaporated to dryness under nitrogen, reconstituted in 1 mL methanol, vortexed, and filtered into autosampler vials.

For determining the LOD and LOQ, 10 replicate injections of the lowest calibration level (1 ppb) were analyzed. The mean and standard deviation of the signal responses were used to calculate LOD and LOQ according to standard validation formulas based on 3 and 9 times the standard deviation, respectively. The LOD values were 0.145 and 0.113 ng/mL for MDMB-4en-PINACA and 5F-MDMB-PICA, respectively, while the corresponding LOQ values were 0.435 and 0.339 ng/mL. The calculated LOD and LOQ concentrations were subsequently prepared and analyzed experimentally to verify detectability and quantifiability under the optimized LC–MS/MS conditions.^[18]^

### 2.6. Linearity and validation procedure

Linearity was evaluated using spiked serum samples at concentrations of 1, 2.5, 5, 10, 25, 50, and 100 ng/mL. Nine independent replicates were analyzed for each concentration. Calibration curves were generated by plotting analyte peak area against nominal analyte concentration. Unweighted linear regression was applied for calibration curve construction, and regression analysis was performed automatically using the LabSolution software. Diazepam-d_5_ was not included in the calibration calculations and served only as a procedural control during sample preparation and instrumental analysis. A correlation coefficient (*r*^2^) ≥ 0.99 was considered indicative of acceptable linearity according to international validation guidelines.^[18]^ In addition to linearity, matrix effect, selectivity, carryover, and stability were evaluated in accordance with European Medicines Agency/Food and Drug Administration bioanalytical validation guidelines. All analytes exhibited bias within ±15% under short-term, long-term, and freeze–thaw conditions, confirming stability across storage and analytical stages. Matrix effect was assessed by comparison of analyte responses in post-extraction fortified serum samples and neat standard solutions. Carryover was evaluated by injection of blank samples immediately following the highest calibration standard. Stability studies included short-term, autosampler, freeze–thaw, and long-term storage assessments.

### 2.7. Accuracy, precision, and recovery

Method accuracy and precision were assessed by analyzing serum samples spiked at 2 concentration levels (10 and 50 ng/mL), each in quintuplicate. Repeatability (RSDr) was determined through analyses conducted on the same day by 2 independent analysts, while within-laboratory reproducibility (RSDwR) was evaluated on different days under similar conditions. The precision of the method was expressed as the relative standard deviation (%RSD). Accuracy was verified by ensuring that recovery values fell within the 70%–120% acceptance range.^[18]^

### 2.8. Statistical analysis

All statistical evaluations for the validation of 5F-MDMB-PICA and MDMB-4en-PINACA quantification were performed using LabSolution software (version 5.97). Analyses focused on the main validation parameters – linearity, accuracy, precision, recovery, and reproducibility – in accordance with recognized international guidelines.

Linearity was assessed using 9 separate serum samples spiked with standard solutions spanning concentrations from 1 to 100 ng/mL. Calibration curves were automatically generated by the software, and the correlation coefficient (*r*^2^) was used as the indicator of fit. A value of *r*^2^ ≥ 0.99 was interpreted as evidence of excellent linearity within the tested range. Precision was expressed as the %RSD for both intraday and inter-day experiments. RSDr was determined from 5 replicate measurements conducted by 2 independent analysts on the same day at 2 fortification levels (10 and 50 ng/mL). RSDwR was calculated from analyses performed under similar conditions but on different days. Accuracy was verified by comparing measured concentrations with the nominal spiked values. The recovery percentage for each fortification level was computed, and results between 70% and 120% were considered satisfactory according to the acceptance criteria. The LOD and LOQ were estimated using the standard deviation of the analytical response and the slope of the calibration curve, following the formulae recommended in international validation protocols. Comparative evaluation of mean recovery, standard deviation, and %RSD across analysts and fortification levels confirmed the robustness of the analytical procedure. All calculations and data outputs satisfied the predetermined acceptance thresholds, demonstrating that the developed method provides reliable and reproducible performance suitable for application in both forensic and clinical toxicology.

## 3. Results

**Figure 1.**
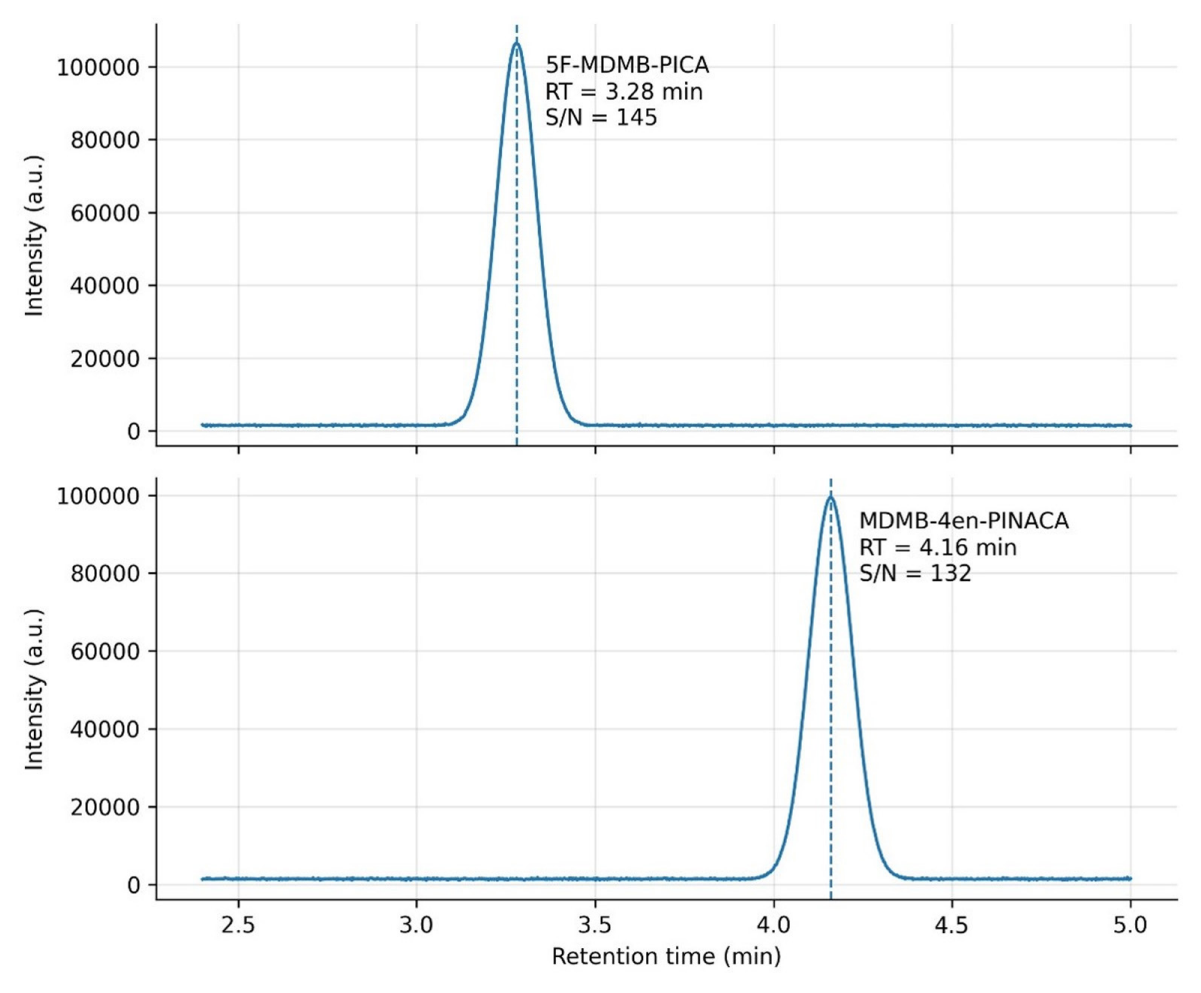
LC–MS/MS chromatograms of 5F-MDMB-PICA and MDMB-4en-PINACA in matrix-matched serum standards showing chromatographic peak separation, retention times, and signal-to-noise ratios. LC–MS/MS = liquid chromatography–tandem mass spectrometry, RT = retention time, S/N = signal-to-noise ratio.

**Figure 2.**
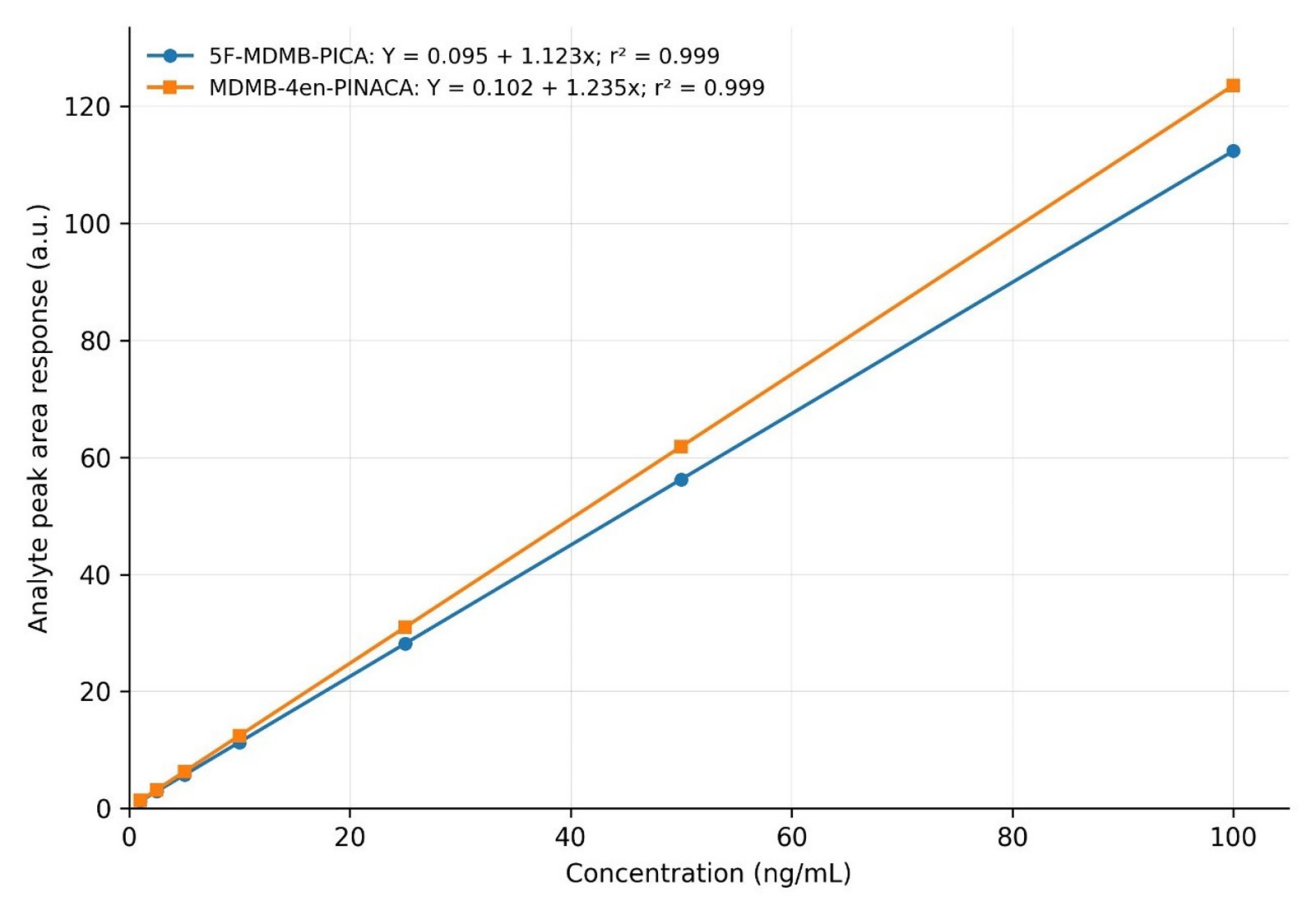
Calibration curves for 5F-MDMB-PICA and MDMB-4en-PINACA obtained from matrix-matched serum standards.

LC–MS/MS chromatograms and calibration data for the target analytes are presented in Figures 1 and 2, and Table 1. Distinct chromatographic peaks were observed for both analytes, with retention times of 3.28 minutes for 5F-MDMB-PICA and 4.16 minutes for MDMB-4en-PINACA. Signal-to-noise ratios were 145 and 132 for 5F-MDMB-PICA and MDMB-4en-PINACA, respectively. Calibration curves demonstrated linear responses across the validated concentration range, yielding correlation coefficients (*r*^2^) of 0.999 for both compounds. The corresponding calibration equations were *Y* = 0.095 + 1.123*x* for 5F-MDMB-PICA and *Y* = 0.102 + 1.235*x* for MDMB-4en-PINACA. The analyte responses increased proportionally with concentration throughout the calibration interval, and the regression parameters remained consistent across replicate analyses (Table 1, Figures 1 and 2).

**Table 1:** Correlation coefficients of calibration curves.

Chemical analyte	Correlation coefficient (*r*^2^)	Calibration curve
5F-MDMB-PICA	0.999	*Y* = 0.095 + 1.123*x*
MDMB-4en-PINACA	0.999	*Y* = 0.102 + 1.235*x*

LC–MS/MS acquisition parameters for the target analytes are presented in Table 2. The quantifier and qualifier transitions monitored for 5F-MDMB-PICA were *m*/*z* 377.2 → 232.1 and *m*/*z* 377.2 → 145.1, respectively, with collision energies of 25 and 35 eV. For MDMB-4en-PINACA, the monitored transitions were *m*/*z* 358.2 → 213.1 and *m*/*z* 358.2 → 145.1, using collision energies of 25 and 35 eV, respectively. All analytes were acquired with a dwell time of 100 ms. The retention times were 3.28 minutes for 5F-MDMB-PICA and 4.16 minutes for MDMB-4en-PINACA. Diazepam-d_5_, used as a procedural control, was monitored using the transition *m*/*z* 290.1 → 198.1 with a collision energy of 20 eV and a retention time of 5.02 minutes (Table 2).

**Table 2:** LC–MS/MS acquisition parameters for target analytes.

Analyte	Precursor ion (*m*/*z*)	Product ion (*m*/*z*)	Collision energy (eV)	Dwell time (ms)	Retention time (min)
5F-MDMB-PICA	377.2	232.1	25	100	3.28
5F-MDMB-PICA (qualifier)	377.2	145.1	35	100	3.28
MDMB-4en-PINACA	358.2	213.1	25	100	4.16
MDMB-4en-PINACA (qualifier)	358.2	145.1	35	100	4.16
Diazepam-d_5_	290.1	198.1	20	100	5.02

*m*/*z*, mass-to-charge ratio.

The assay also showed strong analytical sensitivity. The LOD and LOQ established for both analytes verified that trace levels could be measured accurately (Table 3). Specifically, the LOD values were 0.113 ng/mL for 5F-MDMB-PICA and 0.145 ng/mL for MDMB-4en-PINACA, while the LOQs were 0.339 and 0.435 ng/mL, respectively. These values represent the lowest concentrations that can be quantified with acceptable precision and accuracy, confirming the method’s suitability for detecting minute amounts of the target cannabinoids in serum samples (Table 3).

**Table 3:** LOD and LOQ results.

Chemical analyte	LOD	LOQ
5F-MDMB-PICA (ng/mL)	0.113	0.339
MDMB-4en-PINACA (ng/mL)	0.145	0.435

LOD = limit of detection, LOQ = limit of quantification.

Additional validation experiments demonstrated acceptable analytical performance for both analytes (Table 4). Post-extraction responses were 96.8% for 5F-MDMB-PICA and 94.7% for MDMB-4en-PINACA. The corresponding matrix effect values were −3.2% and −5.3%, with matrix effect coefficients of variation of 5.2% and 6.1%, respectively. Carryover responses accounted for 1.4% of the LOQ response for 5F-MDMB-PICA and 1.8% for MDMB-4en-PINACA. Short-term stability values at 24 hours were 98.5% and 97.2%, while autosampler stability values at 24 hours were 97.4% and 96.8%, respectively. Following repeated freeze–thaw cycles, stability values were 95.9% for 5F-MDMB-PICA and 94.5% for MDMB-4en-PINACA. Long-term stability after 30 days of storage was 94.8% and 93.6%, respectively (Table 4).

**Table 4:** Matrix effect, carryover, and stability parameters of the validated LC–MS/MS method.

Parameter	5F-MDMB-PICA	MDMB-4en-PINACA
Post-extraction response (%)	96.8	94.7
Matrix effect (%)	−3.2	−5.3
Matrix effect CV (%)	5.2	6.1
Carryover (% of LOQ response)	1.4	1.8
Short-term stability (24 h) (%)	98.5	97.2
Autosampler stability (24 h) (%)	97.4	96.8
Freeze–thaw stability (%)	95.9	94.5
Long-term stability (30 days) (%)	94.8	93.6

CV = coefficient of variation, LC–MS/MS = liquid chromatography–tandem mass spectrometry, LOQ = limit of quantification.

The recovery evaluation confirmed that the analytical protocol produced highly consistent and accurate measurements across both analytes. For 5F-MDMB-PICA, the experimentally obtained concentrations at the fortification levels of 10 and 50 ng/mL were 9.8 and 49.6 ng/mL, corresponding to recovery percentages of 98.0% and 99.2%, respectively. Likewise, MDMB-4en-PINACA showed recoveries of 102.0% and 100.2% for the same fortification levels. All recovery values remained well within the acceptable validation limits (70%–120%), verifying that the method meets established international accuracy and reliability criteria (Table 5).

**Table 5:** Recovery test results.

Compound	Measured concentration (ng/mL)	Theoretical concentration (ng/mL)	%Recovery	Within range (70%–120%)
5F-MDMB-PICA	9.8	10.0	98.0	True
	49.6	50.0	99.2	True
MDMB-4en-PINACA	10.2	10.0	102.0	True
	50.1	50.0	100.2	True

The RSDr was conducted to evaluate the short-term precision of the method at 2 fortification levels (10 and 50 ng/mL) and by 2 separate analysts (Table 6). For 5F-MDMB-PICA, average recoveries at 10 ng/mL were 83.7% and 94.0% for Analysts 1 and 2, with corresponding %RSD values of 8.20% and 13.27%. At the 50 ng/mL level, recoveries measured 80.8% and 81.5%, accompanied by %RSD values of 7.48% and 5.78%, respectively. Regarding MDMB-4en-PINACA, the mean recovery percentages were 87.4% and 86.1% at 10 ng/mL, with %RSDs of 5.78% and 5.51%, respectively. At 50 ng/mL, the recoveries increased to 91.7% and 96.4%, yielding %RSDs of 11.46% and 11.18%. All repeatability data remained well within the predefined acceptance criterion of %RSD < 20%, confirming the satisfactory precision of the analytical procedure. The comparable recovery and variability values obtained by both analysts at 2 independent concentration levels underscore the method’s robustness and reliability for quantifying 5F-MDMB-PICA and MDMB-4en-PINACA in serum matrices (Table 6).

**Table 6:** For repeatability (RSDr); % mean recovery, SD, and RSD%.

Chemical analyte	Analysts	Fortification (ng/mL)							
		10 ng/mL				50 ng/mL			
		Mean	SD	%RSD	%Recovery (mean)	Mean	SD	%RSD	%Recovery (mean)
5F-MDMB-PICA	1	8.37	0.69	8.20	83.7	40.38	3.02	7.48	80.8
	2	9.40	1.25	13.27	94.0	40.75	2.35	5.78	81.5
MDMB-4en-PINACA	1	8.74	0.51	5.78	87.4	45.86	5.25	11.46	91.7
	2	8.61	0.47	5.51	86.1	48.19	5.39	11.18	96.4

RSD = relative standard deviation, RSDr = repeatability (intraday precision), SD = standard deviation.

The RSDwR confirmed the consistency of the method when evaluated at 2 concentration levels (10 and 50 ng/mL) and by 2 independent analysts over multiple sessions (Table 7). For 5F-MDMB-PICA, the average recoveries obtained at 10 ng/mL were 82.1% and 77.7%, corresponding to %RSDwR values of 8.17% and 7.51%, respectively. At the higher concentration of 50 ng/mL, recovery percentages increased to 93.5% and 81.6%, with associated RSDwR values of 13.79% and 6.55%. For MDMB-4en-PINACA, the method exhibited similar reproducibility. Mean recovery values at 10 ng/mL were 86.1% for Analyst 1 and 77.7% for Analyst 2, yielding RSDwR values of 4.47% and 7.51%, respectively. At 50 ng/mL, the recoveries were 90.4% and 89.5%, with corresponding RSDwR values of 7.48% and 7.65%. These results collectively verify the precision and stability of the analytical protocol across varying analysts and concentration levels, meeting the reproducibility criteria defined in international validation standards (Table 7).

**Table 7:** For reproducibility (RSDwR); % mean recovery, SD, and RSDwR%.

Chemical analyte	Analysts	Fortification (ng/mL)							
		10 ng/mL				50 ng/mL			
		Mean	SD	% RSDwR	%Recovery (mean)	Mean	SD	% RSDwR	%Recovery (mean)
5F-MDMB-PICA	1	8.21	0.67	8.17	82.1	46.75	6.45	13.79	93.5
	2	7.77	0.58	7.51	77.7	40.80	2.67	6.55	81.6
MDMB-4en-PINACA	1	8.61	0.38	4.47	86.1	45.19	3.38	7.48	90.4
	2	7.77	0.58	7.51	77.7	44.74	3.42	7.65	89.5

RSDwR = within-laboratory reproducibility (inter-day precision), SD = standard deviation.

## 4. Discussion

This study presents a validation of an LC–MS/MS procedure optimized for the quantitative determination of 5F-MDMB-PICA and MDMB-4en-PINACA in human serum. The analytical method demonstrated acceptable linearity, precision, accuracy, and reproducibility. Correlation coefficients of *r*^2^ = 0.999 and low LOD/LOQ values (0.113–0.145 ng/mL and 0.339–0.435 ng/mL, respectively) indicate adequate analytical sensitivity for the quantification of trace concentrations of these synthetic cannabinoids. Recovery rates ranging from 77.7% to 96.4%, together with %RSD values below 15%, were consistent with internationally accepted bioanalytical validation criteria.

Several prior investigations have explored LC–MS/MS-based validation approaches across a range of analytes, establishing the platform’s utility in toxicological analysis.^[19–24]^ Di Francesco et al developed a retention time-based LC–MS/MS strategy for psychoactive substances, emphasizing the role of predictive modeling in enhancing compound identification efficiency.^[19]^ Michely et al proposed a rapid, 1-point calibration LC–MS/MS protocol to quantify 45 drugs and their metabolites, enabling faster toxicity assessment in acute poisoning scenarios.^[20]^ Similarly, Montenarh et al introduced a multi-analyte LC–MS/MS approach for neuroleptic detection across blood, plasma, and serum, demonstrating high sensitivity with LODs as low as 0.1 ng/mL.^[21]^ Shin et al used a scaled-down QuEChERS extraction combined with tandem mass spectrometry to detect multiple pesticide residues in urine with sub-nanogram sensitivity.^[22]^ Rodrigues et al validated a modified micro-QuEChERS method coupled with LC–MS/MS to quantify psychotropic drugs in postmortem blood.^[23]^ Acosta-Dacal et al applied a QuEChERS-based protocol to measure 218 pesticides in soil, highlighting the robustness and flexibility of LC–MS/MS methodology.^[24]^

Only a limited number of studies have applied comparable analytical validation procedures to synthetic cannabinoids in biological specimens. Yeter et al reported an LC–MS/MS method with bidirectional solid-phase extraction for identifying 57 synthetic cannabinoids and their metabolites in blood, confirming its sensitivity at very low concentrations.^[25]^ Krotulski et al investigated newly emerging analogues such as MDMB-4en-PINACA and characterized their receptor-binding potency, raising important toxicological concerns regarding their pharmacological activity.^[26]^ Giorgetti et al expanded the analytical scope by developing a high-throughput LC–MS/MS assay for 182 new psychoactive substances in whole blood, enabling large-scale forensic screening.^[27]^ The present study complements these findings by providing a validated and serum-specific approach for the quantitative detection of 2 high-risk synthetic cannabinoids. The low LOD and LOQ values reported here demonstrate analytical sensitivity that is comparable – or even superior – to that of other published LC–MS/MS methods for psychoactive compounds. Only a limited number of studies have validated LC–MS/MS methods for synthetic cannabinoids in biological specimens, and most have focused on urine or whole blood. The present study extends this analytical framework to serum and optimizes the chromatographic parameters to achieve reliable separation and detection of 5F-MDMB-PICA and MDMB-4en-PINACA with sub-nanogram sensitivity.

When benchmarked against comparable LC–MS/MS assays for synthetic cannabinoids, the current method achieved lower limits of detection and higher recovery rates while maintaining reproducibility below 15%, fulfilling all European Medicines Agency/Food and Drug Administration bioanalytical validation criteria. This performance, combined with serum-specific optimization and dual-analyte quantification, highlights the method’s analytical advancement and its readiness for translation into routine forensic and clinical toxicology.

Validation parameters such as RSDr and RSDwR, which indicate intra- and inter-laboratory precision, have been the focus of numerous studies employing serum or plasma matrices. Montenarh et al performed comparative validations for 33 antidepressants in serum, demonstrating stable matrix effects, consistent recoveries, and acceptable precision ranges.^[28]^ Maxones et al also reported excellent reproducibility using a stable isotope dilution LC–MS/MS assay for fat-soluble vitamins.^[29]^ Likewise, Montenarh et al neuroleptic quantification protocol confirmed the reliability of LC–MS/MS-based therapeutic monitoring by meeting precision thresholds across analytical sessions.^[21]^ These reports collectively align with our present findings, which show RSDr and RSDwR values below 15% for both analytes, validating the method’s reproducibility under routine forensic and clinical conditions. It should be noted that all human serum samples were drug-free and used only as a matrix for analytical spiking. This approach ensures realistic validation of matrix effects while maintaining full compliance with ethical standards. Although real-case serum samples were not included in this study, the validated protocol meets all international analytical standards and is ready for direct translation to forensic and clinical casework once appropriate samples become available.

## 5. Limitations

The method was validated using spiked blank serum samples rather than authentic forensic cases. Future studies will focus on testing this validated protocol on postmortem and clinical specimens to confirm its operational robustness under real-case conditions. Despite the strong analytical performance of the validated LC–MS/MS method, certain limitations should be acknowledged. First, the study was restricted to serum samples; therefore, its applicability to other biological matrices such as urine, saliva, or tissue homogenates was not assessed. Expanding validation across additional matrices would provide a broader understanding of the method’s versatility and potential diagnostic utility. Second, only 2 synthetic cannabinoids – 5F-MDMB-PICA and MDMB-4en-PINACA – were included in the present validation process. The growing diversity of synthetic cannabinoid analogues suggests that additional compounds should be incorporated into future validation studies to ensure comprehensive forensic coverage. Finally, although the method successfully met all acceptance criteria under laboratory conditions, no authentic forensic case samples, external proficiency testing materials, or postmortem specimens were available during the validation process. Consequently, the present work should be regarded primarily as a laboratory-based analytical validation study. Future investigations using real-world forensic and clinical specimens will be necessary to further establish the applicability and robustness of the method under routine casework conditions. Nonetheless, the current study provides a solid analytical foundation for future work focused on expanding detection capabilities for synthetic cannabinoids in clinical and forensic toxicology.

## 6. Conclusions

In conclusion, this study presents a validated LC–MS/MS assay for the quantification of 5F-MDMB-PICA and MDMB-4en-PINACA in human serum. The method demonstrated linearity (*r*^2^ = 0.999), low detection thresholds (LOD: 0.113–0.145 ng/mL; LOQ: 0.339–0.435 ng/mL), and acceptable repeatability and reproducibility in accordance with international bioanalytical validation criteria. These findings indicate that the method can detect and quantify low concentrations of the target synthetic cannabinoids in serum samples. The validated analytical workflow may support future forensic toxicology applications and provides a basis for further studies involving authentic forensic and clinical specimens.

## Acknowledgments

The authors would like to thank Eda Isir (Ankara University Development Foundation High School, Ankara, Turkey) for her valuable assistance during the sample preparation and laboratory processes.

## Author contributions

**Conceptualization:** Murat Akbaba, Aysun Baransel Isir, Süleyman İbili.

**Data curation:** Murat Akbaba, Süleyman İbili, Can Erdem Yiğitsoy.

**Formal analysis:** Murat Akbaba, Aysun Baransel Isir, Süleyman İbili, Can Erdem Yiğitsoy.

**Funding acquisition:** Murat Akbaba.

**Investigation:** Murat Akbaba, Aysun Baransel Isir, Süleyman İbili, Can Erdem Yiğitsoy.

**Methodology:** Aysun Baransel Isir, Süleyman İbili, Can Erdem Yiğitsoy.

**Project administration:** Murat Akbaba.

**Resources:** Aysun Baransel Isir, Can Erdem Yiğitsoy.

**Supervision:** Aysun Baransel Isir.

**Validation:** Aysun Baransel Isir, Süleyman İbili, Can Erdem Yiğitsoy.

**Visualization:** Aysun Baransel Isir, Süleyman İbili, Can Erdem Yiğitsoy.

**Writing – original draft:** Murat Akbaba.

**Writing – review & editing:** Aysun Baransel Isir, Süleyman İbili, Can Erdem Yiğitsoy.
